# MICA and NKG2D variants as risk factors in spondyloarthritis: a case–control study

**DOI:** 10.1038/s41435-018-0044-x

**Published:** 2018-09-04

**Authors:** Marie Fechtenbaum, Judith Desoutter, Gauthier Delvallez, Etienne Brochot, Nicolas Guillaume, Vincent Goëb

**Affiliations:** 1 Department of Rheumatology, Amiens University Medical Center, Amiens, France; 2 Jules Verne University of Picardie, EA HEMATIM Amiens, France; 3 Department of Hematology and Histocompatibility, Amiens University Medical Center, Amiens, France; 4 Department of Virology, Amiens University Medical Center, Amiens, France

**Keywords:** Genetics, Genetic association study

## Abstract

The major histocompatibility complex class I polypeptide-related sequence A (MICA) glycoprotein mediates the activation of the natural killer group 2D receptor (NKG2D) expressed on NK and CD8+ T cells. A methionine or valine at position 129 in exon 3 results in strong (MICA129 met) or weak (MICA129 val) binding to NKG2D. The *MICA A5.1* allele causes a premature stop codon. Various NKG2D polymorphisms are associated with low (*NKC3 C/C* and *NKC4 C/C*) or high (*NKC3 G/G* and *NKC4 T/T*) levels of NK cell cytotoxic activity. In 162 patients with spondyloarthritis (115 with ankylosing spondyloarthritis, 46 with psoriatic arthritis and 1 with reactive arthritis) compared to 124 healthy controls, MICA-129 with methionine allele was more frequent in patients with spondyloarthritis (odds ratio (OR) (95% confidence interval) = 4.84 (2.75‒8.67)), whereas *MICA-129 val/val*, *MICA A5.1* and *NKC3 C/C* variants were less frequent (OR = 0.20 (0.11‒0.37), 0.15 (0.06‒0.36) and 0.24 (0.13‒0.44), respectively). After adjustment for HLA-B*27 status, only *NKC3 C/C* remained linked to spondyloarthritis (adjusted OR = 0.14 (0.06‒0.33)). Homozygosity for *MICA A5.1* is linked to ankylosing spondyloarthritis, and *NKC3 C/C* and *MICA-129 val/val* to psoriatic arthritis. MICA and NKC3 polymorphisms (related to a low NK cell cytotoxic activity) constituted a genetic association with spondyloarthritis.

## Introduction

Spondyloarthritis (SpA) refers to a group of chronic inflammatory diseases with a number of similar features, including axial manifestations (such as sacroiliitis or spondylitis), peripheral manifestations (such as arthritis, enthesitis and dactylitis) and extra-articular manifestations (such as psoriasis, uveitis and inflammatory bowel disease (IBD)) [1]. Ankylosing spondyloarthritis (AS) and psoriatic arthritis (PsA) are the two most prevalent SpA diseases. In young adults, AS and PsA cause irreversible tissue damage and worsen the quality of life [2]. The prevalence of SpA is 0.43% in France, with a sex ratio of 1 [3]. The presence of the well-known genetic marker HLA-B*27 allele in patients with symptoms increases the risk of developing SpA (risk ratio (95% confidence interval (CI)) = 39 (17‒86)), and is one of the Assessment of Spondyloarthritis International Society (ASAS) criteria for diagnosis of this disease [4]. In the general population, the HLA-B*27 allele is found in 1 to 5% of symptom-free individuals. However, this allele accounts for only 20 to 40% of the genetic susceptibility to SpA, suggesting that other genes are significantly involved in the physiopathology of these diseases [5].

On chromosome 6, the gene closest to the *HLA-B* locus is major histocompatibility complex class I chain A related (*MICA*). It codes for a cell stress-inducible glycoprotein [6] that mediates the activation of natural killer group 2D receptor (NKG2D) expression and the associated pro-inflammatory pathway [7–9]. Exons 2–4 of *MICA* correspond to three extracellular domains, and exon 5 corresponds to a transmembrane domain. The *MICA-129* polymorphism (resulting in the replacement of methionine by valine) in exon 3 has been shown to influence NKG2D signaling [10]. Indeed, the presence of methionine vs. valine at position 129 of the α2-heavy chain domain results in strong (*MICA-129 Met/Met*) or weak (*MICA-129 Val/Val*) binding to the NKG2D receptor. Furthermore, seven alleles have been described in exon 5 of *MICA* (from the wild type A4 to A9). The alleles differ with regard to GCT triplet repeat microsatellite polymorphisms. Moreover, the *MICA A5.1* allele has an additional G nucleotide insertion, which creates a premature stop codon and results in the production of truncated transmembrane MICA protein (i.e., lacking the cytoplasmic domain) [11].

NKG2D is an activating receptor from the C-type lectin-like family of transmembrane proteins. It is expressed on natural killer (NK) cells, CD8+ T cells and NK T cells. Eight single-nucleotide polymorphisms (SNPs) are known to be closely associated with differences in the level of cytotoxic activity. In particular, *NKC3 C/C* and *NKC4 C/C* are associated with a low level of NK cell cytotoxicity, whereas *NKC3 G/G* and *NKC4 T/T* are associated with a high level [12].

Contrasting results have been reported for the association between SpA and *MICA* polymorphisms [13–17]. To the best of our knowledge, NKG2D receptor polymorphisms have been studied in some inflammatory diseases (such as rheumatoid arthritis) but not yet in SpA [18].

The primary objective of this study was to determine whether MICA and NKG2D polymorphisms (linked to the cytotoxic activity of NK cells) are associated with a predisposition to SpA (i.e., AS or PsA) and the clinical and radiological features of the disease.

## Results

### Demographic characteristics of the study groups

Patients with SpA (*n* = 162, mean (range) age at diagnosis of SpA: 40 (9–72), 55% male) were compared with healthy control participants (*n* = 90, 63.7% male) (Table 1). The mean (range) disease duration was 8.6 (1‒46) years. The HLA-B*27 allele was observed in 99 patients (61%) and 4 controls (3.2%, *p* = 1.8 × 10^−18^). At diagnosis, 80, 44, 23 and 11% of the patients presented with back pain, arthritis, enthesitis and dactylitis respectively. All patients met ASAS criteria. One hundred and thirty-one patients were ASAS-positive axial SpA: 110 patients had structural damage or sacroiliitis (SI X-ray, computed tomography (CT) scan or magnetic resonance imaging (MRI)) and 21 patients were HLA-B*27 positive associated with more than 2 others symptoms. Thirty-one patients were only ASAS-positive peripheral SpA. In 51 patients who had a SI X-ray, 21 patients (13%) met the modified New York (mNY) criteria. Other patients had SI CT scan or MRI or did not have SI explorations because they had only peripheral symptoms. One hundred and fifteen patients (71%) had AS, 46 (28%) had PsA and 1 patient had reactive arthritis.

**Table 1:** Demographic, clinical and radiological characteristics of patients with spondyloarthritis

Characteristics: *n* = 162 patients	Results
Age at diagnosis, mean (range) (years)	40 (9–72)
Disease duration, mean (range) (years)	8.6 (1–46)
Male, *n* (%)	90 (55)
HLA-B*27, *n* (%)	99 (61)
Back pain, *n* (%)	130 (80)
Arthritis, *n* (%)	72 (44)
Enthesitis, *n* (%)	38 (23)
Dactylitis, *n* (%)	18 (11)
Uveitis, *n* (%)	16 (10)
Psoriasis, *n* (%)	44 (27)
Inflammatory bowel disease, *n* (%)	14 (8)
Sacroiliitis or structural damage, *n* (%)	
• X-ray (*n* = 51)	33 (65)
• X-ray, CT or MRI (*n* = 153)	110 (72)
Erosion on hands or foot X-ray, *n* (%) (*n* = 85)	15 (17)
Ankylosing spondyloarthritis, *n* (%)	115 (71)
Psoriatic arthritis, *n* (%)	46 (28)
Reactive arthritis, *n* (%)	1 (1)

*CT* computerized tomography, *MRI* magnetic resonance imaging

### Frequencies of MICA/NKG2D polymorphisms in the SpA population

The overall observed frequencies of *MICA/NKG2D* polymorphisms are listed in Table 2. The homozygote *MICA 129 Val/Val* was found to protect against the development of SpA (odds ratio (OR) (95% CI) = 0.20 (0.11‒0.37)), whereas the *MICA 129 Met* allele was more prevalent in patients with SpA than in controls and thus constituted a genetic association for SpA (OR = 4.84 (2.75‒8.67)).

**Table 2:** Frequencies of MICA/NKG2D polymorphisms in the SpA population

		*n* (%)		OR (95% CI)	*P*	OR_adj_
		C	P			
*MICA 129*	n	88	162			
	*Met/Met*, *Met/Val*	41 (47)	131 (80)	4.84 (2.75–8.67)	5 × 10^−08^	ns
	*Val/Val*	47 (53)	31 (19)	0.20 (0.11–0.37)	2 × 10^−08^	ns
*MICA A5.1*	*n*	90	161			
	*abs*	29 (32)	85 (53)	2.16 (1.10–4.30)	2 × 10^−05^	ns
	*Hetero*	41 (46)	68 (43)	0.56 (0.32–0.99)	3 × 10^−05^	ns
	*Homo*	20 (22)	9 (6)	0.15 (0.06–0.36)	6 × 10^−05^	ns
*NKC3*	*n*	90	160			
	*C/C*	41 (46)	27 (17)	0.24 (0.13–0.44)	1 × 10^−06^	0.14 (0.06–0.33)
	*G/G*, *G/C*	49 (54)	133 (83)	4.12 (2.33–7.47)	2 × 10^−06^	6.73 (3.04–16.6)
*NKC4*	*n*	124	161			
	*T/T*	6 (5)	8 (5)	1.03 (0.35–3.19)	ns	ns
	*C/C*, *C/T*	118 (95)	153 (95)	0.97 (0.31–2.87)	ns	ns

*C* controls, *P* patients, *OR* odd ratio, *OR*_*adj*_ adjusted odd ratio, *ns* not significant

Heterozygous and homozygous *MICA A5.1* mutations constituted a genetic association with SpA, with ORs of 0.56 (0.32‒0.99) and 0.15 (0.06‒0.36), respectively.

The *NKC-3 C/C* polymorphism was found to protect against SpA (OR = 0.24 (0.13‒0.44)), whereas the presence of a G mutation in *NKC3* constituted a genetic association with SpA (OR = 4.12 (2.33‒7.47)). After adjustment for HLA-B*27 status, these polymorphisms remained significantly associated with protection (OR_adj_ = 0.14 (0.06‒0.33)) and disease risk (OR_adj_ = 6.73 (3.04‒16.6)), respectively.

No significant associations between *NKC4* polymorphisms and SpA were found.

All three protective markers (*MICA 129 Val/Val*, the *MICA A5.1* mutation and the *NKC3 C/C* polymorphism) were found in 2 of the 160 patients with SpA (1%) and in 12 of the 90 controls (13%). This combination protected against SpA (OR = 0.08 (0.01‒0.31); *p* = 0.0001).

In multivariate analysis, before adjustment for the HLA-B*27 status, heterozygous and homozygous *MICA A5.1* mutations, the presence of a G mutation in *NKC3* constituted a genetic association with SpA, with ORs of 0.60 (0.37‒0.95), 0.20 (0.09‒0.45) and 4.51 (2.35‒8.93), respectively, whereas the *NKC3 C/C* polymorphism was found to protect against SpA (OR = 0.22 (0.11‒0.42)). No significant associations between *NKC4* polymorphisms and SpA were found. Given that only *NKC3* status was significant after HLA-B*27 adjustment, we did not perform a multivariate analysis.

### Frequencies of MICA/NKG2D polymorphisms in AS and PsA subgroups

In patients with AS, the *MICA 129 Val/Val* polymorphism, homozygous *MICA A5.1* mutation and the *NKC3 C/C* polymorphism protected against AS (OR = 0.19 (0.102‒0.362), 0.11 (0.03‒0.31) and 0.30 (0.16‒0.56), respectively). After adjustment for HLA-B*27 status, only the homozygote *MICA A5.1* mutation had still a genetic association (OR_adj_ = 0.28 ((0.067‒0.96)).

In patients with PsA, *MICA 129 Val/Val*, the homozygous *MICA A5.1* mutation and the *NKC3 C/C* polymorphism were protective (OR = 0.24 (0.10‒0.55), 0.32 (0.08‒1.02) and 0.11 (0.16‒0.56), respectively). After adjustment for HLA-B*27 status, *MICA 129 Val/Val* and *NKC3 C/C* polymorphisms were still protective factors (OR_adj_ = 0.33 (0.13‒0.76) and 0.09 (0.02‒0.28), respectively).

Any association was found between *MICA 129 Met/Val*, *MICA A5.1*, *NKC3* *C/G*, *NKC4 C/T* polymorphisms and clinical data (Table 3). Indeed, these polymorphisms are not linked to uveitis, psoriasis, inflammatory bowel disease and sacroiliac joint damage in our cohort.

**Table 3:** Associations between MICA/NKG2D polymorphisms and clinical data

		*n* (%)	Clinical data *n* (OR (95% CI))			
			Uveitis	Psoriasis	IBD	SI
*MICA 129* *N* = 162			*N* = 16	*N* = 44	*N* = 14	*N* = 110
	*Met*	131 (80)	16	34	12	93
	*Val/Val*	31 (20)	0 (∞ (0.97–∞))	10 (0.74 (0.29–1.93))	2 (1.45 (0.29–14.93))	17 (2 (0.82–4.28))
*MICA A5.1* *N* = 161			*N* = 16	*N* = 44	*N* = 14	*N* = 109
	*abs*	85 (53)	10	17	9	63
	*Hetero*	9 (4)	0 (∞ (0.20–∞))	4 (0.45 (0.09–2.37))	0 (∞ (0.17–∞))	3 (4.5 (0.92–29.40))
	*Homo*	67 (43)	6 (0.79 (0.37–4.27))	23 (0.55 (0.25–1.17))	5 (1.31 (0.37–5.23))	43 (1.37 (0.66–2.85))
*NKC3 N* = 160			*N* = 16	*N* = 43	*N* = 14	*N* = 109
	*C/C*	27 (17)	4	5	2	19
	*G*	133 (83)	12 (1.74 (0.37–6.45))	38 (0.57 (0.15–1.69))	12 (0.80 (0.08–3.91))	90 (1.13 (0.43–3.24))
*NKC4 N* = 160			*N* = 16	*N* = 43	*N* = 14	*N* = 109
	*T/T*	8 (5)	1	2	2	5
	*C*	153 (95)	15 (1.31 (0.02–11.42))	41 (0.91 (0.09–5.36))	12 (3.86 (0.34–24.95))	104 (0.43 (0.08–2.98))

*IBD* inflammatory bowel disease, *SI* sacroiliac joint damage on X-ray or CT scan or magnetic resonance imaging, *C* cytosine, *G* guanine, *Hetero* heterozygous, *Homo* homozygous, *Met* methionine, *T* thymidine, *Val* valine

## Discussion

Associations between MICA/NKG2D polymorphisms and several immune-mediated diseases have been reported previously. In particular, MICA polymorphisms are associated with inflammatory rheumatic diseases [19], Behçet’s disease [20], IBD1 [21], systemic lupus [22] and ankylosing spondylitis [23, 24]. NKG2D variants have also been linked to rheumatoid arthritis susceptibility and severity [18]. Given the complex gene sequences and protein expression profiles of MICA/NKG2D, studies of the pathogenesis of immune-mediated SpA are essential. To address this issue, we genotyped *MICA129* and *MICA A5.1* polymorphisms, and two NKG2D SNPs (rs1049174 for NKC3 and rs2255336 for NKC4) in patients with SpA and in healthy controls.

Our study population of mainly Caucasian patients was similar to the literature data with regard to age at diagnosis, sex ratio, the frequency of AS vs. PsA and the percentage of patients bearing HLA-B*27 alleles [2, 3]. We had a small number of patients meeting the mNY criteria because the majority of our patient did not have the result of SI X-ray in their data (the result was not available at the time of collecting data and/or the used of MRI is more common than the SI X-ray to diagnose a SpA). However, all patients met ASAS criteria.

We found that *MICA 129 Val/Val* constituted a genetic association with SpA (i.e., both AS and PsA). In the study of Zhou et al. [23] of 1070 American patients with AS (all of whom met the New York modified criteria), the same result was found both before and after adjustment for HLA-B*27 status. We found that *MICA-129 Val/Val* protected against PsA. We also found that this polymorphism constituted a genetic association with X-ray-confirmed sacroiliitis. In view of the link between *MICA129 Val/Val* and low affinity for its receptor, one pathophysiological hypothesis holds that patients with SpA and *MICA129 Val/Val* polymorphism have lower levels of inflammation near their sacroiliac joint and thus suffer less damage from sacroiliitis.

In the study of Zhou et al. [23], heterozygous and homozygous *MICA A5.1* mutations protected against AS in 1070 American patients (OR = 0.35 (0.29–0.42)) and 473 Chinese patients (0.51 (0.39–0.67) [23]. Our present results also showed that heterozygous and homozygous *MICA A5.1* mutations were linked to PsA.

To the best of our knowledge, the association between *NKC3* polymorphisms and SpA has not previously been studied. Here, we found that the presence of a G mutation in *NKC3* was a risk factor for SpA both before and after adjustment for HLA-B*27 status. *NKC3 G/G* status could be used to refine the diagnosis of SpA, particularly when a patient is negative for HLA-B*27 status. Given the association between *NKC3 G/G* and high levels of cytotoxicity [12], it would be interesting to understand the relationship between this polymorphism and severe, aggressive forms of the disease.

It is well established that the *MICA* gene confers susceptibility to SpA [23]; indeed, MICA*007:01 is a significant risk allele for SpA in Caucasian and Han Chinese populations, and MICA*019 is a major risk allele in Chinese patients. Moreover, the *MICA129 met/met* genotype was linked to juvenile SpA (independently of HLA-B*27 status) in Algerian patients [24], rheumatic disease-associated IBD in Spanish patients [25] and systemic lupus erythematosus in Japanese patients [19]. *NKG2D* gene variants have also been linked to rheumatoid arthritis susceptibility and severity [18]. Indeed, the frequencies of the *NKG2D9* A allele and the *NKG2D10* T allele were significantly higher in patients with deformities, whereas the frequencies of the *NKG2D9* G allele and the *NKG2D10* A allele were higher in patients without deformities. The mechanism by which *MICA/NKG2D* polymorphisms protect against inflammatory rheumatic diseases has not been fully elucidated. However, Nielsen et al. [26] showed that fibroblast-like synoviocytes from patients with rheumatoid arthritis (RA-FLS) express many ligands of activating and inhibitory NK cell receptors. Blockade of the interaction between CD94/NGA (an inhibitory receptor) and its ligand HLA-E expressed on RA-FLSs enhanced degranulation of the Nishi human NK cell line [26]. Moreover, blockade of NKG2D in a murine model of collagen-induced arthritis was associated with significant joint protection, relative to control animals [27]. Patients with psoriatic arthritis have elevated expression levels of interleukin-15 and MIC in their affected synovial tissues, and this inflammatory environment enabled NK cell activation and tissue destruction through NKG2D [28].

The present study was performed in a large, representative cohort of patients with SpA with the use of asymptomatic control patient. It constituted a multidisciplinary collaboration between geneticists and rheumatologists. In view of the retrospective, single-center design, our study had some limitations. Furthermore, the PsA subgroup was relatively small (*n* = 46), even though the proportion of patients with PsA was much the same as in other cohorts [3].

Studies of the association between *MICA/NKG2D* polymorphisms and inflammatory diseases have generated conflicting results. Our present results show that *MICA A5.1*, *MICA129 val/val* and *NKC3 C/C* polymorphisms (related to low levels of cytotoxic activity in NK cells) protected against SpA. In contrast, *MICA129 met*, *NKC3 G/G* and *G/C* polymorphisms (related to elevated levels of cytotoxic activity and an inflammatory environment) were risk factors for SpA. Further research must focus on the involvement of MICA/NKG2D signaling in rheumatic disease.

## Methods

### Study participants

We performed a retrospective, single-center study of Caucasian patients with SpA being treated in the Rheumatology Department at Amiens University Hospital (Amiens, France). The procedures were approved by the Nord Ouest II ethics committee, Amiens, France. In accordance with French legislation and the Declaration of Helsinki, all participants gave their prior, written, informed consent to genetic testing. Spondyloarthritis had been diagnosed by an experienced rheumatologist in all cases.

A total of 214 patients with SpA were screened between August 2014 and May 2015. We excluded 52 patients who were lost to follow-up or had not provided written, informed consent. Hence, 162 patients were included in the study. Demographic, clinical, laboratory, radiological and HLA-B*27 genotyping (performed by PCR-SSP: Single Specific Primer-Polymerase Chain Reaction, Bionobis, Paris, France) data were extracted from the patients’ medical records. All the radiological data had been analyzed by the same specialist radiologist at the time of diagnosis. Sacroiliac joint damages were diagnosed on an X-ray, CT scan or MRI of the sacroiliac joint [29, 30]. Erosions were diagnosed on hands and foot X-rays. Retrospectively, patients were classified with mNY and ASAS criteria [29, 30].

### Controls

One hundred and twenty healthy control Caucasian participants were selected from adult hematopoietic stem cell donor registries. In line with the World Marrow Donor Association’s guidelines, none had a history of auto-immune disease, neoplastic disease or thromboembolic events.

### NKG2D and MICA polymorphisms

NKG2D genotyping was performed as described previously [31], using a TaqMan® allelic discrimination method on a 9700-HT real-time polymerase chain reaction (PCR) system (Applied Biosystems, Foster City, CA, USA). An allele-specific fluorogenic oligonucleotide (Applied Biosystems) was used to discriminate between genotypes for each studied pair of alleles. The following two SNPs were probed: (i) rs1049174, featuring a G-C substitution that distinguishes between the high-activity-related HNK1 NKG2D haplotype (G) and the low-activity-related LNK1 (C) haplotype (NKC3); and (ii) rs2255336, featuring a C-T substitution that distinguishes between the HNK2 (T) and LNK2 (C) haplotypes (NKC4). All participants underwent sequence-based typing for MICA. PCR primers were designed to amplify MICA exons 3 and 5, as described previously [31]. Sequencing was performed on an automatic 24-capillary ABI Prism system (Applied Biosystems), and the electropherogram was interpreted using SeqPilot® software (JSI Medical Systems GmbH, Ettenheim, Germany).

### Statistical analysis

Data were quoted as the mean and range (for continuous variables) or the number and percentage (for categorical variables). Univariate logistic regression was used to compare the frequency of polymorphism in patients vs. controls. The odds ratio was adjusted (OR_adj_) for HLA-B*27 status by including this variable in the logistic regression. Each genotype was compared with the others as the reference. Variables with a *p* value < 0.1 in a univariate analysis were included in a multivariate model, where the threshold for statistical significance was set to *p* < 0.05. Associations between clinical and radiological features and polymorphisms were also probed. All analyses were performed with R software (version 3.1).

## References

[1] Baeten D, Breban M, Lories R, Schett G, Sieper J (2013). Are spondylarthritides related but distinct conditions or a single disease with a heterogeneous phenotype?. Arthritis Rheum.

[2] Dougados M, Etcheto A, Molto A, Alonso S, Bouvet S, Daurès JP (2015). Clinical presentation of patients suffering from recent onset chronic inflammatory back pain suggestive of spondyloarthritis: the DESIR cohort. Joint Bone Spine.

[3] Costantino F, Talpin A, Said-Nahal R, Goldberg M, Henny J, Chiocchia G (2015). Prevalence of spondyloarthritis in reference to HLA-B27 in the French population: results of the GAZEL cohort. Ann Rheum Dis.

[4] Bowness P (2015). HLA-B27. Annu Rev Immunol.

[5] Cortes A, Hadler J, Pointon JP, Robinson PC, Karaderi T, International Genetics of Ankylosing Spondylitis Consortium (IGAS (2013). Identification of multiple risk variants for ankylosing spondylitis through high-density genotyping of immune-related loci. Nat Genet.

[6] Visser CJ, Tilanus MG, Tatari Z, van der Zwan AW, Bakker R, Rozemuller EH (1999). Sequencing-based typing of MICA reveals 33 alleles: a study on linkage with classical HLA genes. Immunogenetics.

[7] Raulet DH (2003). Roles of the NKG2D immunoreceptor and its ligands. Nat Rev Immunol.

[8] Colucci F, Di Santo JP, Leibson PJ (2002). Natural killer cell activation in mice and men: different triggers for similar weapons?. Nat Immunol.

[9] Renedo M, Arce I, Montgomery K, Roda-Navarro P, Lee E, Kucherlapati R (2000). A sequence-ready physical map of the region containing the human natural killer gene complex on chromosome 12p12.3-p13.2. Genomics.

[10] Isernhagen A, Malzahn D, Viktorova E, Elsner L, Monecke S, von Bonin F (2015). The MICA-129 dimorphism affects NKG2D signaling and outcome of hematopoietic stem cell transplantation. EMBO Mol Med.

[11] Ota M, Katsuyama Y, Mizuki N, Ando H, Furihata K, Ono S (1997). Trinucleotide repeat polymorphism within exon 5 of the MICA gene (MHC class I chain-related gene A): allele frequency data in the nine population groups Japanese, Northern Han, Hui, Uygur, Kazakhstan, Iranian, Saudi Arabian, Greek and Italian. Tissue Antigens.

[12] Hayashi T, Imai K, Morishita Y, Hayashi I, Kusunoki Y, Nakachi K (2006). Identification of the NKG2D haplotypes associated with natural cytotoxic activity of peripheral blood lymphocytes and cancer immunosurveillance. Cancer Res.

[13] Breban M, Miceli-Richard C, Zinovieva E, Monnet D, Said-Nahal R (2006). The genetics of spondyloarthropathies. Joint Bone Spine.

[14] Mameli A, Cauli A, Taccari E, Scarpa R, Punzi L, Lapadula G (2008). Association of MICA alleles with psoriatic arthritis and its clinical forms. A multicenter Italian study. Clin Exp Rheumatol.

[15] Song GG, Kim JH, Lee YH (2014). Associations between the major histocompatibility complex class I chain-related gene A transmembrane (MICA-TM) polymorphism and susceptibility to psoriasis and psoriatic arthritis: a meta-analysis. Rheumatol Int.

[16] Pollock R, Chandran V, Barrett J, Eder L, Pellett F, Yao C (2011). Differential major histocompatibility complex class I chain-related A allele associations with skin and joint manifestations of psoriatic disease. Tissue Antigens.

[17] Pollock RA, Chandran V, Pellett FJ, Thavaneswaran A, Eder L, Barrett J (2013). The functional MICA-129 polymorphism is associated with skin but not joint manifestations of psoriatic disease independently of HLA-B and HLA-C. Tissue Antigens.

[18] Mariaselvam CM, Tamouza R, Krishnamoorthy R, Charron D, Misra DP, Jain VK (2017). Association of NKG2D gene variants with susceptibility and severity of rheumatoid arthritis. Clin Exp Immunol.

[19] Wang Q, Zhou X (2015). Associations of MICA polymorphisms with inflammatory rheumatic diseases. Open Rheumatol J.

[20] Mizuki N, Ota M, Kimura M, Ohno S, Ando H, Katsuyama Y (1997). Triplet repeat polymorphism in the transmembrane region of the MICA gene: a strong association of six GCT repetitions with Behçet disease. Proc Natl Acad Sci USA.

[21] Orchard TR, Dhar A, Simmons JD, Vaughan R, Welsh KI, Jewell DP (2001). MHC class I chain-like gene A (MICA) and its associations with inflammatory bowel disease and peripheral arthropathy. Clin Exp Immunol.

[22] Yoshida K, Komai K, Shiozawa K, Mashida A, Horiuchi T, Tanaka Y (2011). Role of the MICA polymorphism in systemic lupus erythematosus. Arthritis Rheum.

[23] Zhou X, Wang J, Zou H, Ward MM, Weisman MH, Espitia MG (2014). MICA, a gene contributing strong susceptibility to ankylosing spondylitis. Ann Rheum Dis.

[24] Amroun H, Djoudi H, Busson M, Allat R, El Sherbini SM, Sloma I (2005). Early-onset ankylosing spondylitis is associated with a functional MICA polymorphism. Hum Immunol.

[25] López-Hernández R, Valdés M, Lucas D, Campillo JA, Martínez-Garcia P, Salama H (2010). Association analysis of MICA gene polymorphism and MICA-129 dimorphism with inflammatory bowel disease susceptibility in a Spanish population. Hum Immunol.

[26] Nielsen N, Pascal V, Fasth AER, Sundström Y, Galsgaard ED, Ahern D (2014). Balance between activating NKG2D, DNAM-1, NKp44 and NKp46 and inhibitory CD94/NKG2A receptors determine natural killer degranulation towards rheumatoid arthritis synovial fibroblasts. Immunology.

[27] Andersson AK, Sumariwalla PF, McCann FE, Amjadi P, Chang C, McNamee K (2011). Blockade of NKG2D ameliorates disease in mice with collagen-induced arthritis: a potential pathogenic role in chronic inflammatory arthritis. Arthritis Rheum.

[28] Tang F, Sally B, Ciszewski C, Abadie V, Curran SA, Groh V (2013). Interleukin 15 primes natural killer cells to kill via NKG2D and cPLA2 and this pathway is active in psoriatic arthritis. PLoS One.

[29] Hermann KGA, Baraliakos X, van der Heijde DMFM, Jurik AG, Landewé R, Marzo-Ortega H (2012). Descriptions of spinal MRI lesions and definition of a positive MRI of the spine in axial spondyloarthritis: a consensual approach by the ASAS/OMERACT MRI study group. Ann Rheum Dis.

[30] Lambert RGW, Bakker PAC, van der Heijde D, Weber U, Rudwaleit M, Hermann KG (2016). Defining active sacroiliitis on MRI for classification of axial spondyloarthritis: update by the ASAS MRI working group. Ann Rheum Dis.

[31] Apithy MJ, Charbonnier A, Desoutter J, Diouf M, Morel P, Garçon L (2018). Impact of MICA and NKG2D polymorphisms in HLA-fully matched related and unrelated hematopoietic stem cell transplantation. Bone Marrow Transplant.

